# Poly[tetra-μ-cyanido-*trans*-bis­(di­methyl­formamide-κ*O*)manganese(II)nickel(II)]

**DOI:** 10.1107/S241431462500762X

**Published:** 2025-09-05

**Authors:** Abderezak Addala, Zouaoui Setifi, Joel T. Mague, Fatima Setifi, Mohammed Hadi Al-Douh

**Affiliations:** a Laboratoire de Chimie Ingénierie Moléculaire et Nanostructures (LCIMN) Université Ferhat Abbas Sétif 1 Sétif 19000 Algeria; b Département de Technologie Faculté de Technologie Université 20 Août 1955-Skikda BP 26 Route d'El-Hadaiek Skikda 21000 Algeria; c Department of Chemistry, Tulane University, New Orleans, LA 70118, USA; d Chemistry Department, Faculty of Science, Hadhramout University, Mukalla, Hadhramout, Yemen; Katholieke Universiteit Leuven, Belgium

**Keywords:** crystal structure, solvothermal reaction, manganese(II), nickel(II), cyanide, di­methyl­formamide, polymer

## Abstract

In the crystal of the title compound, [MnNi(CN)_4_(C_3_H_7_NO)_2_]_*n*_, both metal cations lie on sites of 2/*m* symmetry, with the Ni^II^ ion being four-coordinate square-planar and the Mn^II^ ion being six-coordinate octa­hedral. Both coordination spheres are slightly distorted from the ideal shapes. The {C≡N→Mn} unit is distinctly non-linear and all four cyanido ligands on each [Ni(CN)_4_]^2−^ unit coordinate to Mn^II^ ions, leading to the formation of an infinite layer structure. Bond lengths and inter­bond angles are comparable to those in similar dimetallic, cyanido-bridged compounds.

## Abstract 2

In the title compound, both metal atoms lie on sites of 2/*m* symmetry with the Ni^II^ ion four-coordinate square planar and the Mn^II^ ion six-coordinate octa­hedral. Both coordination spheres are slightly distorted from the ideal geometries. The {C≡ N→Mn} unit is distinctly non-linear and all four cyano ligands on each [Ni(CN)_4_]^2−^ unit are coordinated to Mn^II^ ions, leading to the formation of an infinite layer structure.

## Graphical abstract

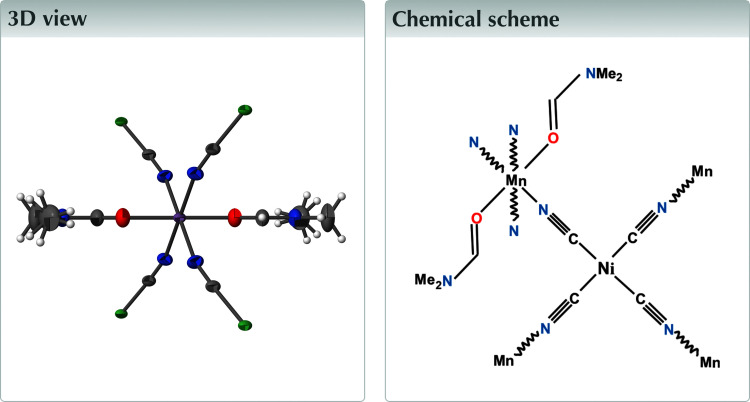

## Structure description

The design and synthesis of polymeric cyanido-bridged metal complexes has received much attention in recent years due to inter­esting magnetic (Benmansour *et al.*, 2012 ▸; Atmani *et al.*, 2008 ▸) and spin-crossover phenomena (Benmansour *et al.*, 2010 ▸; Setifi *et al.*, 2014 ▸), and luminescence (Addala *et al.*, 2019 ▸). These polymeric metal complexes are formed by metal–metal or metal–ligand–metal bridge connections in one, two or three periodicities (Benmansour *et al.*, 2007 ▸, 2009 ▸). Mono-periodic coordination compounds based on cyanido complexes are being intensively studied at present due to their inter­esting magnetic properties (Setifi *et al.*, 2009 ▸, 2013 ▸).

Cyanido­metallate anions show various shapes, *e.g.* linear as in [*M*(CN)_2_]^−^ (*M* = Au or Ag), trigonal as in [Cu(CN)_3_]^2−^, tetra­hedral as in [Cd(CN)_4_]^2−^, square-planar as in [*M*(CN)_4_]^2−^ (*M* = Ni, Pd or Pt), and octa­hedral as in [*M*(CN)_6_]^3−^ (*M* = Fe, Co, Cr or Mn). The diamagnetic, square-planar anions [*M*(CN)_4_]^2−^, where *M* = Ni, Pt, and Pd, are ideal building blocks for the construction of coordination polymers, due to the ability of the four cyanide groups to connect to other metal cations, and thus build up mol­ecular assemblies, either heterometallic or homometallic, with different periodicities (Alexandrov *et al.*, 2015 ▸).

As a part of our continuing research on the synthesis and characterizations of polymeric cyano­carbanion or cyanido­metallate complexes, we report herein the crystal structure of a layered heterometallic polymer, [MnNi(CN)_4_(C_3_H_7_NO)_2_]_*n*_ (I), based on the [Ni(CN)_4_]^2−^ moiety as ligand.

**Figure 1:**
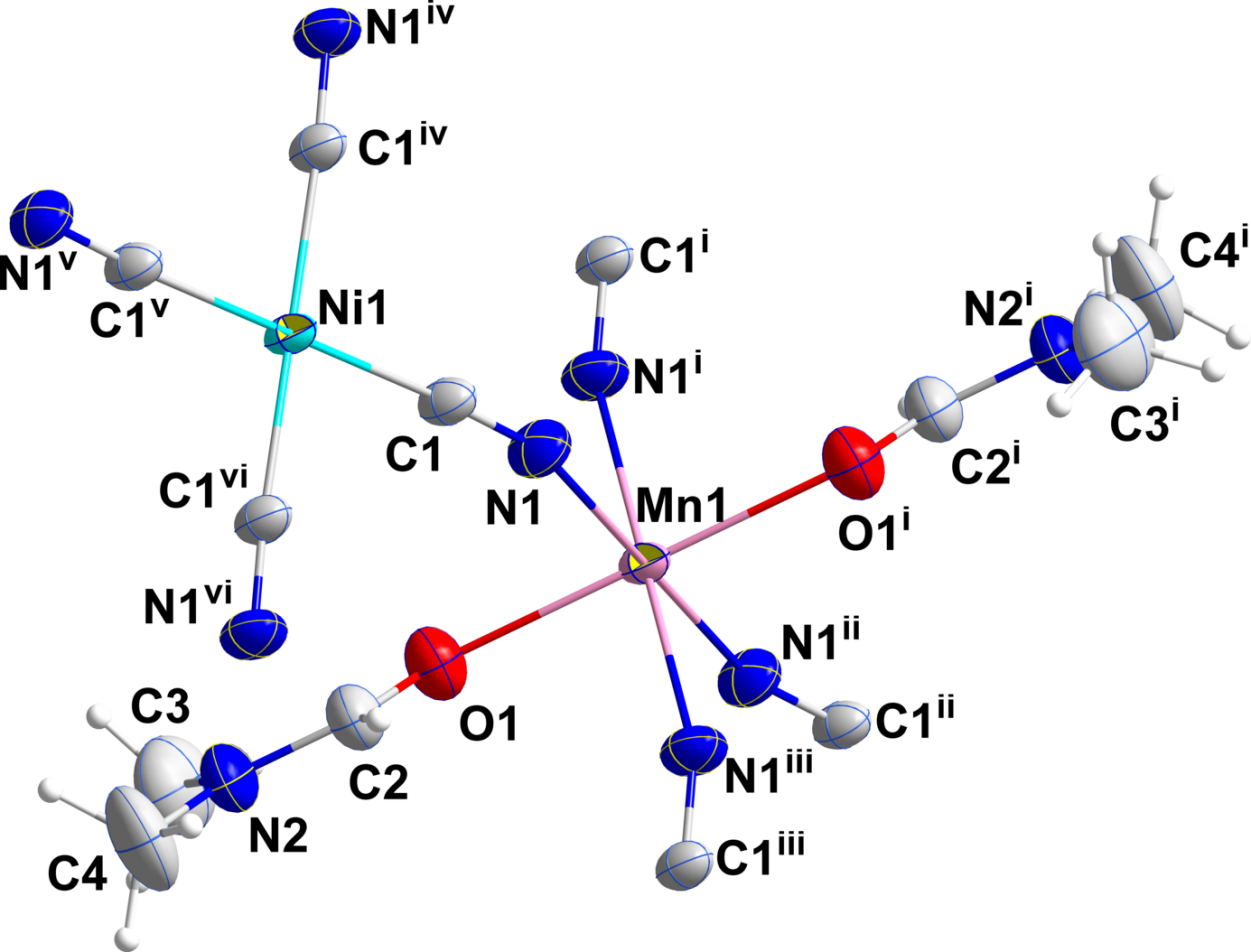
Perspective view of the coordination spheres of Mn1 and Ni1 with labelling scheme and 50% probability ellipsoids [symmetry codes: (i) −*x* + 1, *y*, −*z* + 1; (ii) −*x* + 1, −*y* + 1, −*z* + 1; (iii) *x*, −*y* + 1, *z*; (iv) *x*, −*y* + 2, *z*; (v) −*x* + 1, −*y* + 2, −*z* + 2; (vi) −*x* + 1, *y*, −*z* + 2].

**Figure 2:**
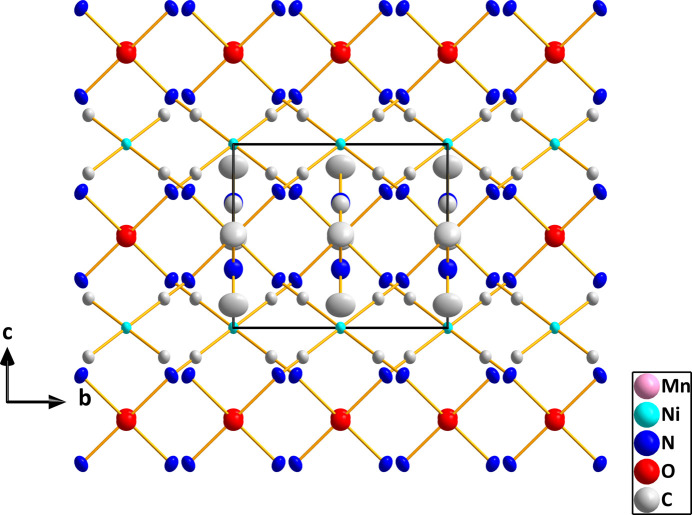
Packing viewed along the *a*-axis direction with hydrogen atoms omitted for clarity.

**Figure 3:**
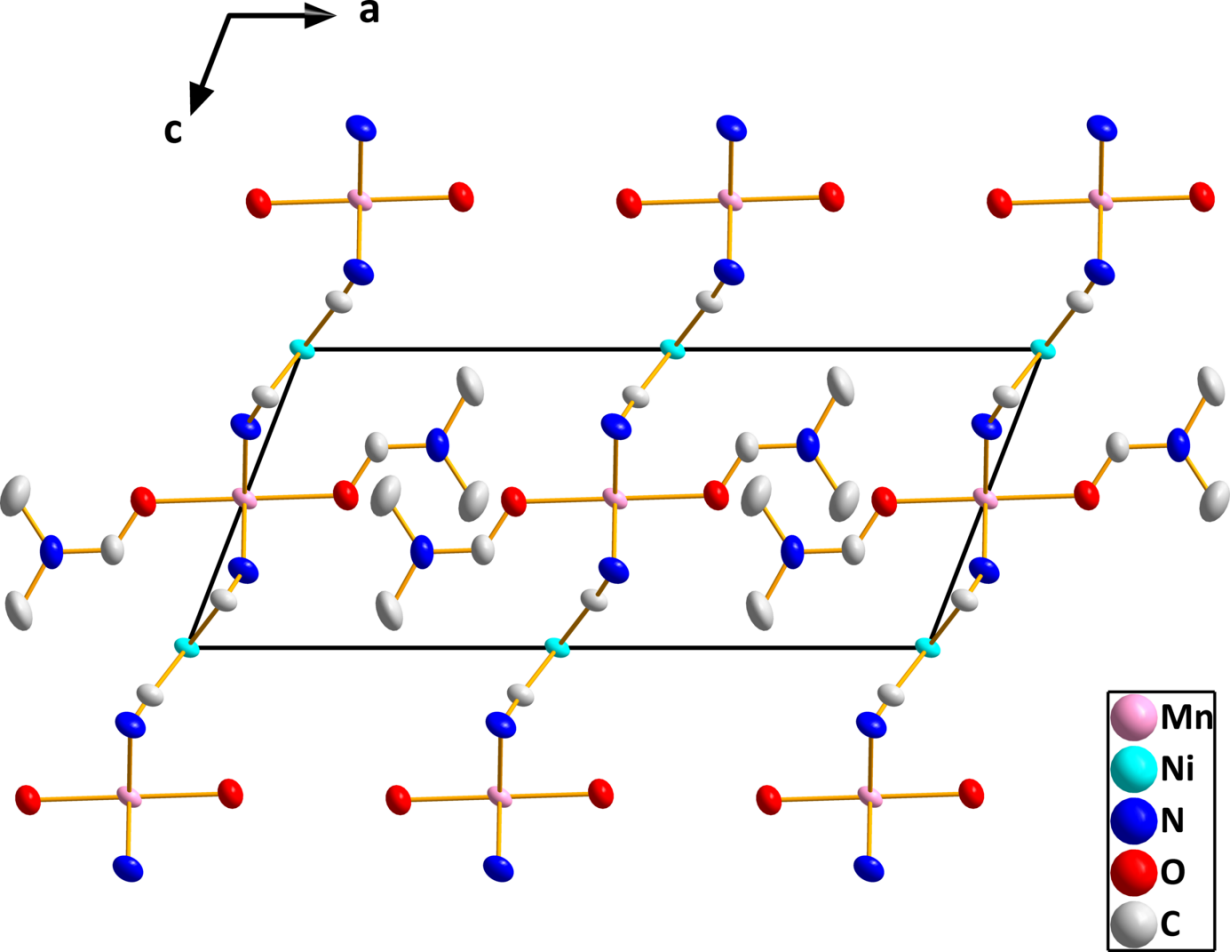
Packing viewed along the *b*-axis direction with hydrogen atoms omitted for clarity.

In (I), both metal ions sit on sites of 2/*m* symmetry, thus requiring pairs of *trans* ligands to be exactly 180° apart. However, the coordination environments of Ni1 and Mn1 are slightly distorted from idealized square-planar and octa­hedral, respectively (Fig. 1 ▸). For Ni1, this is the result of the C1—Ni1—C1^iv^ angle being 91.36 (5)° and the C1—Ni1—C1^vi^ angle 88.64 (5)°, and for Mn1 having N1—Mn1—N1^iii^ and N1—Mn1—N1^i^ angles of 91.17 (5) and 88.83 (5)°, respectively. Additionally, the axial-equatorial angles for Mn1 are not 90° with the O1—Mn1—N1 and O1—Mn1—N1^i^ angles being 88.04 (3) and 91.96 (3)°, respectively (symmetry codes are given in Fig. 1 ▸). The Ni1—C1 distance of 1.8595 (9) Å is comparable to those found in several cyanido-bridged Ni/Cd complexes (Yang, 2020 ▸; Yuge *et al.*, 1995 ▸) and in [Ni(en)_2_Ni(CN)_4_]_*n*_ (Černák *et al.*, 1988 ▸) while the Mn1—N1 distance of 2.2219 (9) Å is longer than the corresponding Ni—N distance in [Ni(en)_2_Ni(CN)_4_]_*n*_ [2.126 (4) Å] reflecting the larger radius of the Mn^II^ ion. As with the compounds mentioned above, the link between the metals is not linear as the C1≡N1→Mn1 angle is 157.79 (8)°. This is towards the lower end of the range found in the compounds cited above. In contrast to those compounds, the manganese cation in the title compound does not contain bidentate ligands and thus all four cyanido ligands on each nickel ion form bridges to manganese ions. The result is a slightly corrugated layer structure with the layers parallel to the *bc* plane as is best illustrated in Fig. 3. The layers are associated primarily by van der Waals inter­actions between the *N*,*N*-di­methyl­formamide ligands on manganese (Figs. 2 ▸ ▸ and 4 ▸).

## Synthesis and crystallization

A mixture of manganese(II) chloride (13 mg, 0.1 mmol) and dipotassium nickel(II) tetra­cyanide (24 mg, 0.1 mmol), *N*,*N*-di­methyl­formamide (12 ml) and water (6 ml) was sonicated for 20 min. Then the reaction mixture was transferred to a Teflon-lined stainless steel reactor and placed in the oven. Subsequently, the temperature was kept 375 K for 3 days. After cooling to room temperature at a rate of 10 K h^−1^, light blue-shaped crystals of (I) were obtained.

## Refinement

Crystal and refinement details are presented in Table 1 ▸.

## Supplementary Material

Crystal structure: contains datablock(s) I. DOI: 10.1107/S241431462500762X/vm4072sup1.cif

Structure factors: contains datablock(s) I. DOI: 10.1107/S241431462500762X/vm4072Isup2.hkl

CCDC reference: 2482894

Additional supporting information:  crystallographic information; 3D view; checkCIF report

## Figures and Tables

**Figure 4:**
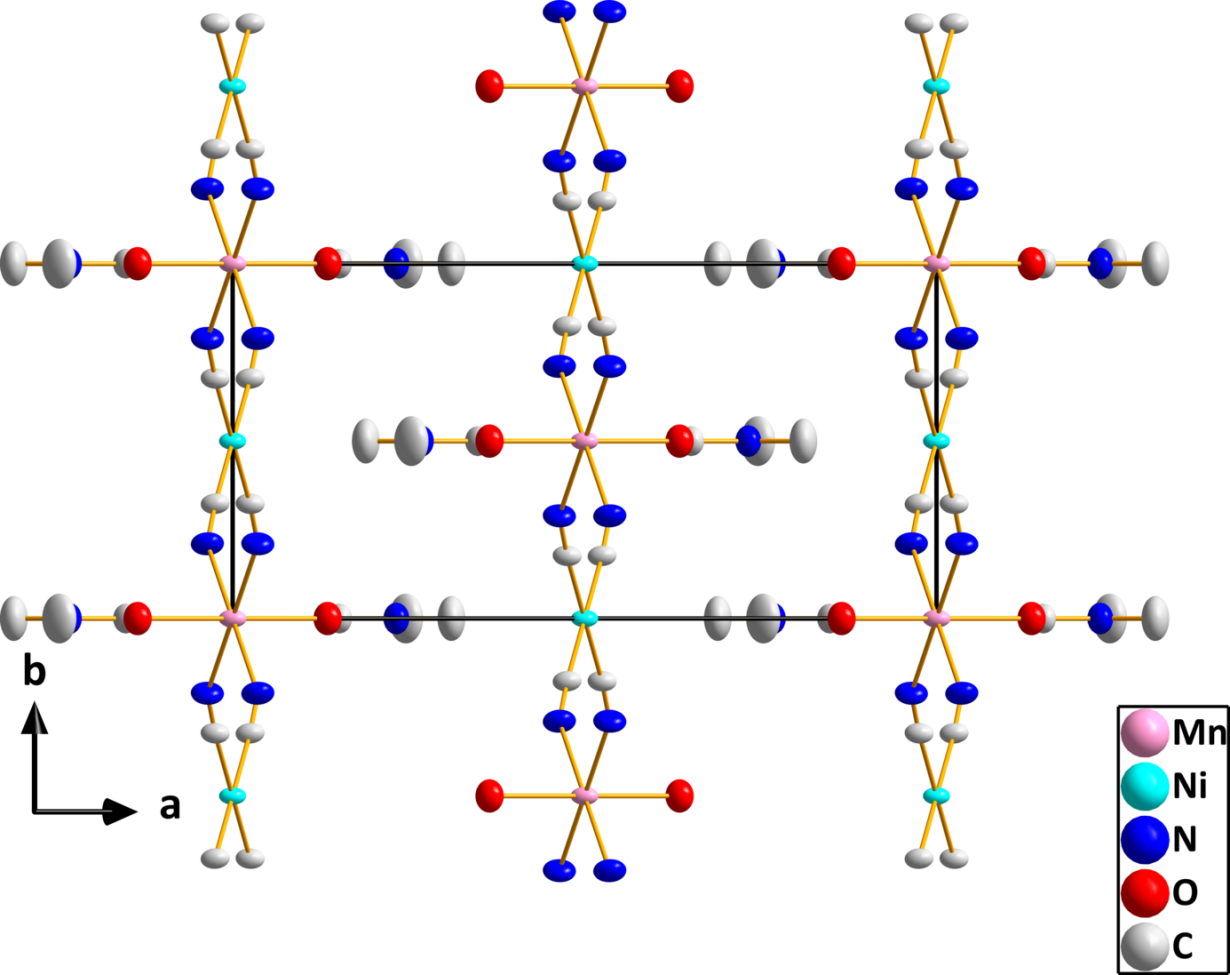
Packing viewed along the *c*-axis direction with hydrogen atoms omitted for clarity.

**Table 1:** Experimental details

Crystal data	
Chemical formula	[MnNi(CN)_4_(C_3_H_7_NO)_2_]
*M*_r_	363.92
Crystal system, space group	Monoclinic, *C*2/*m*
Temperature (K)	300
*a*, *b*, *c* (Å)	16.0430 (4), 7.5345 (2), 6.9185 (2)
β (°)	111.162 (1)
*V* (Å^3^)	779.88 (4)
*Z*	2
Radiation type	Mo *K*α
μ (mm^−1^)	2.03
Crystal size (mm)	0.32 × 0.21 × 0.11

Data collection	
Diffractometer	Bruker D8 Venture dual source
Absorption correction	Multi-scan (*SADABS*; Krause *et al.*, 2015 ▸)
*T*_min_, *T*_max_	0.587, 0.768
No. of measured, independent and observed [*I* > 2σ(*I*)] reflections	17239, 2005, 1595
*R*_int_	0.055
(sin θ/λ)_max_ (Å^−1^)	0.834

Refinement	
*R*[*F*^2^ > 2σ(*F*^2^)], *wR*(*F*^2^), *S*	0.023, 0.065, 1.03
No. of reflections	2005
No. of parameters	59
H-atom treatment	H atoms treated by a mixture of independent and constrained refinement
Δρ_max_, Δρ_min_ (e Å^−3^)	0.36, −0.28

Computer programs: *APEX3* and *SAINT* (Bruker, 2016 ▸), *SHELXT2014/5* (Sheldrick, 2015*a* ▸), *SHELXL2019/2* (Sheldrick, 2015*b* ▸), *DIAMOND* (Brandenburg & Putz, 2012 ▸), *SHELXTL* (Sheldrick, 2008 ▸) and *publCIF* (Westrip, 2010 ▸)08.

## full crystallographic data

### Poly[tetra-µ-cyanido-trans-bis(dimethylformamide-κO)manganese(II)nickel(II)] Crystal data

**Table:**

[MnNi(CN)_4_(C_3_H_7_NO)_2_]	*F*(000) = 370
*M**_r_* = 363.92	*D*_x_ = 1.550 Mg m^−^^3^
Monoclinic, *C*2/*m*	Mo *K*α radiation, λ = 0.71073 Å
*a* = 16.0430 (4) Å	Cell parameters from 7456 reflections
*b* = 7.5345 (2) Å	θ = 2.9–25.7°
*c* = 6.9185 (2) Å	µ = 2.03 mm^−^^1^
β = 111.162 (1)°	*T* = 300 K
*V* = 779.88 (4) Å^3^	Block, light blue
*Z* = 2	0.32 × 0.21 × 0.11 mm

### Poly[tetra-µ-cyanido-trans-bis(dimethylformamide-κO)manganese(II)nickel(II)] Data collection

**Table:**

Bruker D8 Venture dual source diffractometer	2005 independent reflections
Radiation source: micro-source	1595 reflections with *I* > 2σ(*I*)
Graphite monochromator	*R*_int_ = 0.055
φ and ω scans	θ_max_ = 36.4°, θ_min_ = 3.0°
Absorption correction: multi-scan (SADABS; Krause *et al.*, 2015)	*h* = −26→22
*T*_min_ = 0.587, *T*_max_ = 0.768	*k* = −12→12
17239 measured reflections	*l* = −11→11

### Poly[tetra-µ-cyanido-trans-bis(dimethylformamide-κO)manganese(II)nickel(II)] Refinement

**Table:**

Refinement on *F*^2^	Primary atom site location: dual
Least-squares matrix: full	Secondary atom site location: difference Fourier map
*R*[*F*^2^ > 2σ(*F*^2^)] = 0.023	H atoms treated by a mixture of independent and constrained refinement
*wR*(*F*^2^) = 0.065	*w* = 1/[σ^2^(*F*_o_^2^) + (0.0323*P*)^2^ + 0.1831*P*] where *P* = (*F*_o_^2^ + 2*F*_c_^2^)/3
*S* = 1.03	(Δ/σ)_max_ < 0.001
2005 reflections	Δρ_max_ = 0.36 e Å^−^^3^
59 parameters	Δρ_min_ = −0.28 e Å^−^^3^
0 restraints	

### Poly[tetra-µ-cyanido-trans-bis(dimethylformamide-κO)manganese(II)nickel(II)] Special details

**Table:**

Geometry. All esds (except the esd in the dihedral angle between two l.s. planes) are estimated using the full covariance matrix. The cell esds are taken into account individually in the estimation of esds in distances, angles and torsion angles; correlations between esds in cell parameters are only used when they are defined by crystal symmetry. An approximate (isotropic) treatment of cell esds is used for estimating esds involving l.s. planes.
Refinement. Refinement of F^2^ against ALL reflections. The weighted R-factor wR and goodness of fit S are based on F^2^, conventional R-factors R are based on F, with F set to zero for negative F^2^. The threshold expression of F^2^ > 2sigma(F^2^) is used only for calculating R-factors(gt) etc. and is not relevant to the choice of reflections for refinement. R-factors based on F^2^ are statistically about twice as large as those based on F, and R- factors based on ALL data will be even larger. H-atoms attached to carbon were placed in calculated positions (C—H = 0.95 - 0.98 Å). All were included as riding contributions with isotropic displacement parameters 1.2 - 1.5 times those of the attached atoms.

### Poly[tetra-µ-cyanido-trans-bis(dimethylformamide-κO)manganese(II)nickel(II)] Fractional atomic coordinates and isotropic or equivalent isotropic displacement parameters (Å^2^)

**Table:**

	*x*	*y*	*z*	*U*_iso_*/*U*_eq_	Occ. (<1)
Mn1	0.500000	0.500000	0.500000	0.02335 (6)	
Ni1	0.500000	1.000000	1.000000	0.02217 (6)	
N1	0.53584 (7)	0.71064 (12)	0.74098 (14)	0.03595 (18)	
O1	0.36505 (8)	0.500000	0.5107 (2)	0.0438 (3)	
N2	0.26747 (9)	0.500000	0.6793 (3)	0.0445 (3)	
C1	0.52487 (6)	0.82343 (12)	0.84090 (14)	0.02759 (16)	
C2	0.34842 (10)	0.500000	0.6720 (3)	0.0390 (3)	
H2	0.3947 (14)	0.500000	0.810 (3)	0.047*	
C3	0.18920 (14)	0.500000	0.4909 (4)	0.0762 (8)	
H3A	0.162294	0.615663	0.469772	0.114*	0.5
H3B	0.147054	0.414072	0.502401	0.114*	0.5
H3C	0.206332	0.470264	0.375482	0.114*	0.5
C4	0.25393 (18)	0.500000	0.8761 (5)	0.0788 (9)	
H4A	0.310851	0.500230	0.987816	0.118*	
H4B	0.221091	0.395852	0.885356	0.118*	0.5
H4C	0.220892	0.603918	0.885129	0.118*	0.5

### Poly[tetra-µ-cyanido-trans-bis(dimethylformamide-κO)manganese(II)nickel(II)] Atomic displacement parameters (Å^2^)

**Table:**

	*U*^11^	*U*^22^	*U*^33^	*U*^12^	*U*^13^	*U*^23^
Mn1	0.03685 (13)	0.01552 (10)	0.02312 (11)	0.000	0.01736 (10)	0.000
Ni1	0.03538 (11)	0.01429 (9)	0.01987 (10)	0.000	0.01364 (8)	0.000
N1	0.0524 (5)	0.0257 (4)	0.0353 (4)	−0.0015 (3)	0.0224 (4)	−0.0080 (3)
O1	0.0385 (5)	0.0532 (8)	0.0453 (6)	0.000	0.0218 (5)	0.000
N2	0.0322 (6)	0.0483 (9)	0.0580 (9)	0.000	0.0223 (6)	0.000
C1	0.0400 (4)	0.0200 (3)	0.0258 (3)	−0.0013 (3)	0.0155 (3)	−0.0013 (3)
C2	0.0314 (6)	0.0427 (9)	0.0466 (9)	0.000	0.0187 (6)	0.000
C3	0.0370 (9)	0.096 (2)	0.0857 (18)	0.000	0.0105 (10)	0.000
C4	0.0566 (13)	0.118 (3)	0.0802 (19)	0.000	0.0475 (14)	0.000

### Poly[tetra-µ-cyanido-trans-bis(dimethylformamide-κO)manganese(II)nickel(II)] Geometric parameters (Å, º)

**Table:**

Mn1—O1^i^	2.1929 (11)	N2—C3	1.447 (3)
Mn1—O1	2.1929 (11)	N2—C4	1.454 (3)
Mn1—N1^i^	2.2219 (9)	C2—H2	0.98 (2)
Mn1—N1^ii^	2.2219 (9)	C3—H3A	0.9600
Mn1—N1^iii^	2.2219 (9)	C3—H3B	0.9600
Mn1—N1	2.2219 (9)	C3—H3C	0.9600
Ni1—C1	1.8595 (9)	C3—H3A^ii^	0.9600
Ni1—C1^iv^	1.8596 (9)	C3—H3B^ii^	0.9600
Ni1—C1^v^	1.8596 (9)	C3—H3C^ii^	0.9600
Ni1—C1^vi^	1.8596 (9)	C4—H4A	0.9600
N1—C1	1.1484 (12)	C4—H4B	0.9600
O1—C2	1.2373 (19)	C4—H4C	0.9600
N2—C2	1.3177 (19)		
			
O1^i^—Mn1—O1	180.0	N2—C2—H2	112.0 (12)
O1^i^—Mn1—N1^i^	88.04 (3)	N2—C3—H3A	109.5
O1—Mn1—N1^i^	91.96 (3)	N2—C3—H3B	109.5
O1^i^—Mn1—N1^ii^	91.96 (3)	H3A—C3—H3B	109.5
O1—Mn1—N1^ii^	88.04 (3)	N2—C3—H3C	109.5
N1^i^—Mn1—N1^ii^	88.83 (5)	H3A—C3—H3C	109.5
O1^i^—Mn1—N1^iii^	88.04 (3)	H3B—C3—H3C	109.5
O1—Mn1—N1^iii^	91.96 (3)	N2—C3—H3A^ii^	109.47 (3)
N1^i^—Mn1—N1^iii^	91.17 (5)	H3A—C3—H3A^ii^	130.4
N1^ii^—Mn1—N1^iii^	180.0	H3B—C3—H3A^ii^	27.0
O1^i^—Mn1—N1	91.96 (3)	H3C—C3—H3A^ii^	84.8
O1—Mn1—N1	88.04 (3)	N2—C3—H3B^ii^	109.47 (9)
N1^i^—Mn1—N1	180.00 (4)	H3A—C3—H3B^ii^	27.0
N1^ii^—Mn1—N1	91.17 (5)	H3B—C3—H3B^ii^	84.8
N1^iii^—Mn1—N1	88.83 (5)	H3C—C3—H3B^ii^	130.4
C1—Ni1—C1^iv^	180.0	H3A^ii^—C3—H3B^ii^	109.5
C1—Ni1—C1^v^	91.36 (5)	N2—C3—H3C^ii^	109.47 (12)
C1^iv^—Ni1—C1^v^	88.64 (5)	H3A—C3—H3C^ii^	84.8
C1—Ni1—C1^vi^	88.64 (5)	H3B—C3—H3C^ii^	130.4
C1^iv^—Ni1—C1^vi^	91.36 (5)	H3C—C3—H3C^ii^	27.0
C1^v^—Ni1—C1^vi^	180.0	H3A^ii^—C3—H3C^ii^	109.5
C1—N1—Mn1	157.79 (8)	H3B^ii^—C3—H3C^ii^	109.5
C2—O1—Mn1	124.57 (11)	N2—C4—H4A	109.5
C2—N2—C3	120.80 (18)	N2—C4—H4B	109.5
C2—N2—C4	121.23 (19)	H4A—C4—H4B	109.5
C3—N2—C4	117.97 (19)	N2—C4—H4C	109.5
N1—C1—Ni1	176.37 (8)	H4A—C4—H4C	109.5
O1—C2—N2	124.81 (17)	H4B—C4—H4C	109.5
O1—C2—H2	123.2 (12)		
			
Mn1—O1—C2—N2	180.000 (1)	C4—N2—C2—O1	180.000 (1)
C3—N2—C2—O1	0.000 (1)		

Symmetry codes: (i) −*x*+1, −*y*+1, −*z*+1; (ii) *x*, −*y*+1, *z*; (iii) −*x*+1, *y*, −*z*+1; (iv) −*x*+1, −*y*+2, −*z*+2; (v) *x*, −*y*+2, *z*; (vi) −*x*+1, *y*, −*z*+2.
